# Parents’ and Childcare Workers’ Perspectives Toward SARS-CoV-2 Test and Surveillance Protocols in Pre-school Children Day Care Centers: A Qualitative Study Within the German Wü-KiTa-CoV Project

**DOI:** 10.3389/fmed.2022.897726

**Published:** 2022-04-29

**Authors:** David Gierszewski, Peter Konstantin Kurotschka, Maike Krauthausen, Willi Fröhlich, Johannes Forster, Franziska Pietsch, Andrea Streng, Viktoria Rücker, Julia Wallstabe, Katrin Hartmann, Thomas Jans, Geraldine Engels, Marcel Romanos, Peter Heuschmann, Christoph Härtel, Oliver Kurzai, Johannes Liese, Ildikó Gágyor

**Affiliations:** ^1^Department of General Practice, University Hospital Wuerzburg, Wuerzburg, Germany; ^2^Institute for Hygiene and Microbiology, University of Wuerzburg, Wuerzburg, Germany; ^3^Department of Pediatrics, University Hospital Wuerzburg, Wuerzburg, Germany; ^4^Institute of Clinical Epidemiology and Biometry, University of Wuerzburg, Wuerzburg, Germany; ^5^Clinic and Policlinic for Child and Adolescent Psychiatry, Psychosomatics and Psychotherapy, University Hospital Wuerzburg, Wuerzburg, Germany; ^6^Clinical Trial Center Wuerzburg, University Hospital Wuerzburg, Wuerzburg, Germany; ^7^Leibniz Institute for Natural Product Research and Infection Biology—Hans-Knoell-Institute, Jena, Germany

**Keywords:** parent, childcare worker, child day care centers, child preschool, public health surveillance, COVID-19 testing, qualitative research, interview (MeSH)

## Abstract

**Background:**

Feasibility of surveillance through continuous SARS-CoV-2 testing in pre-school children and childcare workers (CCWs) to prevent closure of day care centers (DCCs) was proven in the Wü-KiTa-CoV study. The purpose of this study was to describe the factors that facilitate or hinder the implementation of continuous SARS-CoV-2 testing from the perspective of parents and CCWs involved in the study.

**Methods:**

A total of 148 semi–structured telephone interviews, repeated before and after the implementation of the surveillance protocols, were conducted with parents and CCWs belonging to the DCCs involved in Wü-KiTa-CoV and analyzed using qualitative content analysis.

**Results:**

Five main topical categories that influences implementation of surveillance protocols for SARS-CoV-2 in DCCs emerged: Generating valuable knowledge, Impact on daily life, Communication and information, Children’s wellbeing and the Sense of security. Smooth integration in daily routines, quickly delivered test results, and efficient communication and information between the study team and the participants were identified as factors that had a positive impact on implementation. To ensure children’s wellbeing, the introduction of non-invasive testing procedures such as saliva testing, parental involvement to motivate, and prepare children for the procedure, the creation of a child-friendly environment for testing, and use of child-friendly explanations were considered critical. The surveillance was found to increase the sense of security during the pandemic. Conversely, reliability of tests in the surveillance protocols, low participation rates, non-transparent communication, the need to travel to testing sites, fear of quarantine in case of positive test results, concerns about higher workloads, the fear of unpleasant feelings for children, their young age, and changing test teams were considered as hindering factors.

**Conclusion:**

This qualitative study of parents of children in day care and DCC staff under surveillance through continuous testing for SARS-CoV-2 in nine German DCCs identified several factors that facilitate or hinder its implementation. These should be considered when planning screening interventions to prevent the spread of SARS-CoV-2 or other infectious diseases in pre-school children DCCs.

## Introduction

In many countries worldwide, since March 2020, the SARS-Cov-2 pandemic went along with unprecedented limitations of public life in the effort to prevent and contain viral spreading. In Germany, these limitations included the closure of schools and preschool-children day care centers (DCCs) during the epidemic peaks (1). Several studies showed negative effects of DCC closures on children’s and adults’ physical and mental health, e.g., leading to lack of physical activity, poor nutrition, increased body weight, and mental health problems (2–6). For families, closure of childcare facilities is challenging as it could lead to loss of working days and salary, contributing to higher levels of anxiety and psychological distress, which are risk factors for parenting related exhaustion, parental violence and child abuse (7, 8). Therefore, it is a public health priority avoiding closures of DCCs during epidemic peaks while preventing the spread of the infection (9). Recently, a non-randomized controlled trial that aimed to evaluate the feasibility, acceptability and efficacy of four different testing and surveillance protocols in children, parents and childcare workers (CCWs) was conducted in nine DCCs in Wuerzburg, Bavaria, from October 2020 to March 2021. The study found that the surveillance protocols tested were well accepted by CCWs, parents and children and mathematical modeling indicated that regular testing is able to minimize the spread of SARS-CoV-2 in DCCs (10). It is recommended that user perspectives are considered when implementing such comprehensive measures (11). Therefore, the aim of the qualitative study was to gain insights in the perspectives of those who were directly responsible for childcare, namely parents and CCWs belonging to the DCCs in which the surveillance protocols were implemented. By analyzing the perspectives of study participants, as well as of individuals refusing to participate, we aimed to uncover factors that may facilitate or hinder the implementation of infectious disease surveillance protocols in DCCs during epidemic outbreaks.

## Materials and Methods

### Study Design, Participant Selection, and Setting

This qualitative study was embedded in Wü-KiTa-CoV, a non-randomized feasibility study to test four different surveillance modules in children and CCWs in nine DCCs. Children and CCWs were tested either *via* mid-turbinate nasal swabbing by trained test teams twice weekly (module 1), or once weekly (module 2), or by self-sampled mouth-rinsing fluid twice weekly (i.e., saliva testing, module 3). While these three modules involved continuous testing, module 4 involved testing only in case of symptoms *via* oropharyngeal swabbing. Surveillance protocols were conducted between October 2020 and March 2021. Overall, 57% of the children and 82% of the CCWs participated in this study (10). While these interventions were carried out, we conducted repeated semi-structured interviews, before and after the implementation of the surveillance protocols, with parents and CCWs by employing a qualitative study design. Participants were identified through a survey that was handed out to all 812 parents and 182 CCWs of the nine participating DCCs in the Wü-KiTa-Cov study. With the aim to represent a wide range of perspectives, among those who agreed to be interviewed, parents and CCWs were selected *via* purposive sampling, taking age, gender, the implemented surveillance protocol, DCC, and their role in childcare (parent or CCW) into account. In addition, a random sample of non-participants in the surveillance protocols were interviewed to collect also the most skeptical viewpoints toward their implementation.

### Data Collection

We conducted repeated interviews in two time periods, before (Late-September—Early-October 2020) and after the implementation of the Wü-KiTa-CoV interventions (Mid-February—Mid-March 2021). This was done to collect information about parents’ and CCWs’ perspectives on and the experiences of the surveillance protocols during their implementation, and to enrich the data (12). Five researchers (DG, WF, MK, AH, and MS) conducted the interviews in German *via* telephone. The interview guide is available in Supplementary Tables 1, 2. To answer the research question of the present study only answers to the leading questions showed in Table 1 were analyzed.

**TABLE 1:** Leading questions and prompts.

	Leading question(s)	Prompts
**First interview period**	*What are your expectations regarding the surveillance protocols?*	*What else would you like to see?* *Where do you see possible problems?*
**Second interview period**	*How did you perceive the surveillance protocols used?*	*Where have difficulties arisen?* *What effects did the surveillance protocols have had*…*:* …*on the day care center?* …*on you personally?* …*on your children?* *How do you estimate an extension of the surveillance protocols used?*

### Data Analysis

Interviews were audio-recorded and transcribed verbatim and continued until data saturation was achieved, defined as the time point when no new themes and perspectives of parents or CCWs emerged in subsequent interviews. All transcribed interviews were de-identified by one researcher (DG). Data analysis was performed according to the principles of qualitative content analysis by Kuckartz using MAXQDA 2020 (13, 14).

Two researchers (DG and WF) read the transcribed interviews from the first and second interview period extensively. They performed line-by-line coding independently of each other to inductively identify main topical categories and sub-categories and develop a category system, while intertwining the two-step coding process based on Kuckartz (13). The consensus on categories and conceptual links among them was reached *via* team meetings at each of the three iterations of data coding. In a further step, two independent researchers, at that time naïve to the analysis (JA, AH) applied the previously developed code-system to the raw data for verification. In a further step, investigator triangulation among DG, WF and two other authors with a different professional background (PK and IG), was used to select and reinterpret the previously defined categories while ensuring that the final ones embraced the full depth of the data. This final stage of the analysis entailed renaming and grouping main topical categories and sub-categories in sense of the research question and several discussions in a multidisciplinary team.

### Ethics and Reporting Standard

Informed consent was obtained in written form, confirmed at the first contact (*via* telephone), and audiotaped. The institutional review boards of the University of Wuerzburg, Germany, approved this study (in-house protocol n. 182/20-me), which is reported in compliance with the Consolidated Criteria for Reporting Qualitative Research (COREQ) guideline (15).

## Results

**FIGURE 1:**
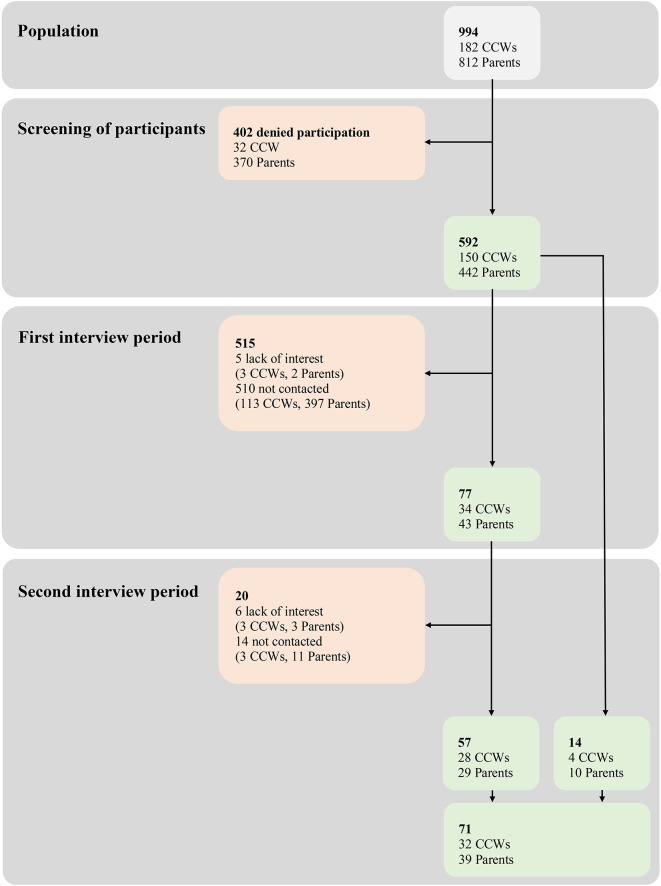
Flow of participants throughout the study with reasons for inclusion/exclusion. Light gray, parents and CCWs belonging to the nine DCCs involved in Wü-KiTa-CoV and screened for interest in being interviewed; light green, parents and CCWs included; light red, parents and CCWs excluded (with reasons).

Among the 994 eligible participants (812 parents and 182 CCWs), 592 (442 parents and 150 CCWs) agreed to be contacted by the study team to be interviewed. Thereafter, interviewers contacted potential participants consecutively throughout the study period. During the first period of data collection, out of 82 contacted people, 77 (34 CCWs and 43 parents) were interviewed, of which 57 (28 CCWs and 29 parents) also took part in a second interview. While six participants (three CCWs and three parents) dropped out (lack of interest) during the second period of data collection, 14 (3 CCWs and 11 parents) were not contacted for reasons of purposive sampling. Instead, 14 new participants (four CCWs and 10 parents) were recruited during this second period of data collection. Figure 1 represents the flow chart of participant recruitment with reasons for inclusion/exclusion, and Table 2 the participants’ characteristics.

**TABLE 2:** Participants’ characteristics.

	First interview period (*n* = 77)		Second interview period (*n* = 71)	
	CCWs: *n* (%)	Parents: *n* (%)	CCWs: *n* (%)	Parents: *n* (%)
**Gender**				
Male	9 (11.7)	14 (18.2)	9 (12.7)	14 (19.7)
Female	25 (32.5)	29 (37.7)	23 (32.4)	25 (35.2)
**Age group (years)**				
18–30	15 (19.5)	3 (3.9)	11 (15.5)	2 (2.8)
31–40	6 (7.8)	33 (42.9)	6 (8.5)	30 (42.3)
41–50	6 (7.8)	7 (9.1)	7 (9.9)	7 (9.9)
51–65	7 (9.1)	0 (0)	8 (11.3)	0 (0)
**Surveillance Module**				
1. Biweekly nasal swab	5 (6.5)	8 (10.4)	5 (7.0)	7 (9.9)
2. Weekly nasal swab	5 (6.5)	8 (10.4)	5 (7.0)	7 (9.9)
3. Biweekly saliva testing	9 (11.7)	11 (14.3)	7 (9.9)	7 (9.9)
4. On-demand oropharyngeal swab	15 (19.5)	16 (20.8)	15 (21.1)	18 (25.4)
**Participation in surveillance protocols**				
Participants	34 (44.2)	39 (50.6)	31 (43.7)	28 (39.4)
Non-participants	0 (0)	4 (5.2)	1 (1.4)	11 (15.5)
**Total**				
	34 (44.2)	43 (55.8)	32 (45.1)	39 (54.9)

For the first interview period, the duration of the interviews ranged from 5 to 30 min (mean = 12 min). For the second period, the duration ranged from 7 to 41 min (mean = 18 min).

Five main topical categories emerged from the analysis that influenced, in the view of participants, the implementation of surveillance protocols for COVID-19 in DCCs. These factors were classified according to whether they were perceived to facilitate or to hinder the implementation of the interventions (Table 3). In Supplementary Table 3, a broad selection of quotes representing categories and subcategories can be found.

**TABLE 3:** Facilitating and hindering factors of the implementation of the Wü-KiTa-CoV surveillance protocols in DCCs from the perspective of participants.

Facilitating factors	Hindering factors
**Generating valuable knowledge**.	
Generate new data Avoiding closures of DCCs Information for policy makers Understand the role of children in the pandemic Understand the impact of the pandemic on children	Skepticism about generating valuable knowledge Doubts on reliability of tests Low participation rates in the study Other testing possibilities
**Impact on daily life**	
Predictable workload Smooth integration in daily routines at the DCCs and at home Quickly delivered test results	Fear of increased workload Need to travel to test centers for PCR testing Fear of quarantines in case of positive test results Carelessness of the testing
**Communication and information**	
Transparent communication Engagement with critical participants Cooperation on equal terms Prompt, brief and thorough information about the surveillance protocols	No possibility of consent withdrawal of sample utilization Too much information about the surveillance protocols Not being informed about the overall results of the surveillance protocols
**Children’s wellbeing**	
Testing in the home environment Tests performed next to a parent Motivation through parents Non-invasive procedures (i.e., saliva tests) Age adequate/Child-friendly explanations Child-friendly testing environment	Fear of development of an aversion to the DCC and medical procedures Young age of the child Fear of unpleasant feelings Invasive procedures (i.e., mid-turbinate nasal swab) Changing test teams
**Sense of security**	
Protocols as sensitive indicators of epidemic outbreaks Sense of protection at work Less fear to transmit the infection to other people Having someone to discuss concerns in case of symptoms Opportunity to get tested timely without complications Opportunity to get tests for household members	Fear of increased risk of infections due to alternating testing staff

### Generating Valuable Knowledge

Parents as well as CCWs felt positive and open-minded toward the opportunity to contribute to knowledge and understanding of several aspects of the pandemic’s management, as well as toward the surveillance protocols. They perceived new data and information to be useful to limit the spread of SARS-CoV-2 infections. Study participants expressed their hope that the study might generate standardized procedures, which might help to avoid extensive DCC closures, to raise awareness in the population, and to aid policy makers in taking appropriate decisions.

“And yes, people might then be more cautious about the pandemic. Aren’t so careless about it. And I think that because of such actions, they might actually think about it.” (CCW 1, female, interview period 1).

Another aspect that emerged was the need of a better understanding of the role of children in the spread of the infection and the impact of the pandemic on their wellbeing as expressed by a parent.

“On the other hand, [by implementing such studies] a little more attention is paid to how this shutdown and this pandemic affect the children.” (Parent 1, female, interview period 2)

Alongside this optimistic attitude, the analysis revealed also some skepticism. At the beginning of the surveillance protocols, some participants questioned the intervention’s ability to generate valuable findings. Participants were also concerned about the reliability of the tests used in the study. While the importance of investigating diagnostic validity was emphasized, doubts were expressed concerning false negative results, not receiving test results in time to avoid the spread of the infection, and the usefulness of rapid serological tests.

“I am just not entirely sure whether a COVID-19 disease can actually be diagnosed accurately using this test. I also believe that there is a relatively high number of unreported cases, or false negative results.” (Parent 2, female, interview period 2)

Parents, as well as CCWs expressed the concern whether the study, because of the low participation rate in some DCCs, would result in relevant findings. A mandatory participation in the surveillance protocols was mentioned as a possible solution to this problem.

“I mean, if you could introduce mandatory actions in all facilities in a sensitive way … no idea, excluding non-participants from the DCC or something like that, then this would maybe at least in the context of the DCC increase the awareness of parents and children.” (CCW 2, male, interview period 1)

Finally, during the study period, test stations became increasingly accessible to the general population. Therefore, according to a CCWs’ coordinator the advantages of the surveillance protocols decreased over time.

“(…) but I wouldn’t see it as a special benefit anymore (…). Simply because, thank God, (…) the whole test situation has changed. It was a sort of luxury, so to speak, that you could test yourself so quickly, but that’s no longer the case.” (CCW 3, female, interview period 2)

### Impact on Daily Life

CCWs initially feared an increased workload associated with the participation in the study interventions, subsequently described as a recurring ritual with a predictable effort, which could easily be incorporated in the daily routines and the pedagogical processes. Moreover, some CCWs felt relieved for not being responsible for the test procedures.

“(…) [It] had little [negative] impact on our everyday pedagogical work. It was just a returning ritual.” (CCW 4, female, interview period 2)

Participants described some aspects of the surveillance protocols (e.g., traveling to the test station, and having additional appointments) as challenging and time consuming for families. Another concern was that the intervention could force CCWs to stay at home while waiting for test results, which could affect the willingness to implement the surveillance protocols. After the intervention, however, it was noticed that the test results were delivered quickly enough to avoid long absences from work.

“I expect that there may be difficulties in actually implementing this: when there are three colleagues in one group [of children] and two of them have cold symptoms [and] one gets tested, that the second one maybe doesn’t want to leave the third alone, feeling like ‘who should be responsible for the group’.” (CCW 5, male, interview period 1)

In addition, parents as well as CCWs feared that positive test results could lead to quarantines or temporary closures of the DCC, which may affect the participants’ personal or professional life negatively. Consequently, some participants stated that they would not be tested voluntarily again. Furthermore, some CCWs, referring to the surveillance protocols in which parents had to carry out tests autonomously, reported episodes in which the parents forgot to carry out the procedures or did not take the surveillance protocols seriously.

“I noticed that some colleagues gurgled with children because their parents either didn’t make it at home or forgot it.“ (CCW 6, female, interview period 2)

### Communication and Information

A transparent communication process and a relationship on equal terms between the Wü-KiTa-CoV study team, the parents, and CCWs involved in the study was described as a way to build trust and strengthen the cooperation. Participants expressed the need to engage with critical participants to dispel doubts or to overcome differences. In this context, some participants assumed that they could not withdraw their consent to usage of their samples for detection of additional respiratory pathogens other than SARS-CoV-2, although this did not correspond to the informed consent.

“(…) When you signed up for it, it said somewhere in the documents that the samples provided would also be used for other purposes. And honestly, I didn’t think that was so good, that first of all you didn’t have the opportunity to contradict that, maybe not everyone would like that, and that you didn’t even know about what would happen with these samples later on. So that I found something like ‘I see, okay.’ (laughs) A bit non-transparent maybe.” (Parent 4, female, interview period 2)

Both parents and CCWs perceived it as relevant to receive thorough information about the study in due time. Concerning study procedures parents and CCWs felt mostly well and timely informed, although a sense of being overwhelmed by the big amount of information was reported, alongside with a wish for more briefness.

“(.) so that it is more compact, on single sheet in A4 format, summarized very briefly, in two sentences, what do you want with this study? What happens when?” (CCW 7, female, interview period 1)

Throughout the intervention, some parents reported to be unaware of how and when they would receive any feedback on the study results (i.e., incidence and immunization rates in the DCC, effectiveness of the different surveillance protocols), while they perceived this as an outcome of the study they would like to be informed about it.

“And I just don’t know, I mean, I don’t know how effective that is either. I also don’t know if we receive an overview of how things went in the other facilities or with the other tests.” (Parent 5, female, interview period 2)

### Children’s Wellbeing

Participants reflected on the wellbeing of the children participating in the surveillance protocols. Both, CCWs and parents were concerned that children would develop an aversion to the DCC or medical procedures as a result of regular mid-turbinate swabs. They also perceived a younger age of the child as a barrier to the feasibility and acceptability of some specific procedures (saliva testing, mid-turbinate swab).

“I am now in the nursery for children aged 1 to 3 (…) most kids just can’t spit it out or are not able to keep it for those 10 seconds in their mouth. Therefore, whether the study is feasible then. But I think older children can do it.” (CCW 8, female, interview period 1)

As a result, some parents denied their children’s participation in the surveillance protocols, even if, in some cases, parents changed their mind afterward, as they realized that regular tests were much less invasive than they expected them to be.

“We consciously, *consciously*, decided against having this nose swabs done weekly (.). But afterwards (.) we would have done it differently. Now that I know it wasn’t that harmful.” (Parent 6, female, interview period 2)

Parents perceived that several factors could play a role to foster the children’s acceptability: performing the tests in the home environment, a parent being next to the child during the sample collection, the child being motivated by parents, using non-invasive procedures, such as saliva tests, giving thorough and child-friendly explanations with possible beforehand practices.

“(…) that I can just do it myself at home [referring to salivary tests]. (…) Because my children are very sensitive and fearful. So, they might even do it voluntarily, but I think it’s easier in the home environment.” (Parent 2, female, interview period 1)

“I just explained to him that it was like taking a booger out of his nose.” (Parent 7, female, interview period 1)

At the same time, parents and CCWs criticized that the test team changed almost every time and that every team carried out the tests differently, whereas a regular team would have been helpful for children to build up trust. In this sense, CCWs acknowledged that the study teams showed appreciation for the children’s efforts in participating in the surveillance protocols, and in creating a child-friendly setting through small gifts, such as stamps or stickers. In the view of the participants, this led children to get better accustomed to the interventions and even proud to take over a social responsibility.

“I think that the children experience themselves as somebody who is doing something for the community. And that they are proud.” (CCW 9, female, interview period 2)

### Sense of Security

Participants perceived the surveillance protocols as an intervention that made them feel safe. The continuous testing was recognized as an early and reliable indicator of changes in the infection process, which could be acted upon to prevent further spreading. The CCWs felt protected at their workplace. Additionally, both parents and CCWs reported to be able to maintain social contacts with less fear of transmitting the infection.

“I basically found it good, the testing (…), because we had (…) at least once per week the verification, regarding (…) one family member, if there was an infection. And this is how I always saw it in the end, that if one of us somehow was infected with COVID, then it would most probably relatively fast spread within the whole family, because we are all pretty close here, like in a house or a household, so to say.” (Parent 8, male, interview period 2)

The analysis revealed that several factors contributed to this perception. Emphasis was placed on always having a contact person to discuss concerns in case of suspected symptoms, especially during the flu-season, as well as the opportunity to get tested timely and to get tests for family or household members easily.

“We were then even able to have our [older] children who were at school tested at the DCC. That was great.” (Parent 10, female, interview period 2).

At the same time, CCWs were concerned about the fact that test teams moved from one DCC to another, and thus could potentially introduce the infection.

“(…) That the doctors and the teams that come in make the children sick. Because you don’t know where they were before.” (CCW 10, male, interview period 1)

## Discussion

This study identified factors that might influence the implementation of surveillance protocols in pre-school children DCCs based on experiences made by parents and CCWs during the pandemic. Smooth integration of testing protocols in daily routines, quickly delivered test results, and efficient communication and information between the study team and the participants were identified as factors that had a positive impact on the implementation. Establishing non-invasive testing procedures, such as saliva tests, involving parents to motivate and prepare children in advance to the procedure, as well as creating a child-friendly environment for testing were seen as crucial to ensure children’s wellbeing. Overall, the implementation of the surveillance protocols was found to increase the sense of security during the pandemic. Conversely, concerns regarding the reliability of tests, low participation rates, the need to travel to testing sites, changing test teams, fear of quarantines in case of positive results, and concerns about higher workloads were perceived as factors that may hinder implementation. Furthermore, both parents and CCWs were concerned about unpleasant feelings that children might experience due to mid-turbinate swabs, as well as the testing of very young children.

W*ellbeing of the children* was considered the most important outcome in our study. The quantitative analyses in Wü-KiTa-CoV showed that the drop-out rate of children with more invasive procedures (mid-turbinate swabs) was significantly higher when compared to those who underwent less-invasive procedures (saliva tests) (10). The present analysis is in line with this finding: parents believing that the procedure would be challenging for their child choose not to participate in the intervention. Considering that saliva tests have already shown to be highly accurate in detecting SARS-CoV-2 (16–18), our findings strengthen with insightful patient-centered data the existing guideline recommendations to perform saliva tests, instead of more invasive procedures, in children under 12 years of age (19). Besides non-invasive procedures, a child-friendly environment, involvement of parents to motivate and prepare children in advance, and test teams with an appreciative attitude toward children were seen as facilitating factors to implement the surveillance protocols in young children. This is in line with previous studies that explored parents’ perspectives and children’s compliance to medical procedures (20, 21). Our participants mentioned that the children felt “proud” for taking over the social responsibility of being tested, and, as previously reported (21), were “always happy” to be rewarded with small gifts. The frequent change of test teams was seen as a factor that made it difficult for children to build trust. These considerations are consistent with previous studies on attachment relationships in children (22).

Study participants reported to be glad to contribute in *generating knowledge*. In their view, this may help to avoid further extensive closures and enhance the understanding of the role of children in the spread of the infection and the impact of the pandemic on the children. These findings are compatible with a recent review on public attitudes toward COVID-19 testing, which found that a benefit of testing was to generate data that may contribute to scientific research and to appropriate policy decisions to manage the pandemic (23). Similarly, a study conducted among students and staff at the University of Nottingham found that scientific interest was motivating in voluntarily testing (24).

In our study, concerns were raised about the diagnostic accuracy of the antibody tests employed and the utility of testing protocols, particularly with regard to prevent the spread of infection. A qualitative report on public understanding of antibody testing in the UK found that participants were uncertain whether someone who tested positive for antibodies could become re-infected or transmit the virus to others. Authors concluded that if such tests are widely offered, *information* should be given to provide clarity about the meaning of the test results and resulting behaviors (25). Similarly, our analysis found that brief and timely information about the surveillance protocols and transparent and easy *communication* with the test teams helps to build trust and to encourage participation and thus were perceived to be important and helpful in implementing the intervention. In a study of participants’ use of and preferences on different types of information provision, Jenkins et al. found that a high level of trust in medical staff might be a reason for not seeking more information about the study (26). In a case-study Enria et al. showed that an approach to participants that entails active and inclusive dialogue, rather than top-down communications, helped to build up trust in the trial teams and, therefore, engagement and participation (27). Consistently, the opportunity for participants to discuss with the teams on site emerged as a factor capable to enable trustful relationships in our study.

Some of our participants were unaware if and when they would have access to the study results, while at the same time they stated knowing the results would be important. There is a general agreement among participants and researchers that research results should always be shared (28, 29), although there is no one-size-fit-all solution that gives a definitive answer to how, when and to what extend this should be done (30). Interestingly, according to a survey, only one-third of participants with previous experience of being enrolled in a study stated to be provided with the study results (28). In the Wü-KiTa-CoV study (10), dissemination of study results comprised an advertisement on the study-website and E-Mails sent to the DCCs’ managers. This was not captured by the evaluation study reported here, as interviews were conducted before the dissemination of results.

We found that the implementation of the Wü-KiTa-CoV interventions gave CCWs a *sense of security* at work, especially in modules 1, 2, and 3, where tests were carried out by a dedicated team and on a regular basis on site. This is compatible with previous studies (31) and our quantitative analyses (10), in which the perceived sense of security was significantly higher among parents of children involved in regular testing rather than in on-demand testing. Moreover, on site testing was experienced as rapidly integrated in the workflow of CCWs (another predictable “daily routine”), and described as reassuring. Furthermore, participants reported to always have someone to talk to about their concerns in case of suspected symptoms. These findings are relevant, as the absence of support and reassurance was found to be associated with burnout and anxiety in educational personnel (32). In addition, parents and CCWs perceived a fast and simple access to COVID-19 tests for themselves and their household members as reassuring. However, it should be considered that since the Wü-KiTa-CoV interventions were carried out, the access to tests increased in all high-income countries (33), and tests provided within the study for study participants alone may not be reassuring enough to motivate people to participate in such surveillance protocols. Other factors, such as the fear of increased workload or increased risk of infection due to moving test teams, as well as the fear of resulting quarantines may hinder the implementation and lower the acceptance of surveillance protocols, and have to be taken into account when implementing such programs in pre-school children DCCs.

### Strengths and Limitations

To the best of our knowledge, this is the first qualitative study that explores the perspectives of parents and CCWs toward SARS-CoV-2 surveillance protocols in children DCCs, highlighting facilitating and hindering factors for the implementation of such interventions.

The results were gained through selection of participants *via* purposive sampling, to take into account a wide range of different perspectives. In particular, we were able to interview participants from both sexes, different age and role in childcare, who participated in all the implemented protocols. In addition, we interviewed also non-participants in the surveillance protocols, as we were interested to uncover also skeptical viewpoints. Only 6 out of 77 participants who were interviewed during the first period of data collection did not want to be interviewed at the second period: this very low number of participants lost at follow-up indicates a high acceptance of our telephone interviews. To further strengthen our study, researchers with different professional backgrounds (DG: a sociologist, MK: health psychologist, WF: a medical doctoral student, PKK, and IG: academic primary care physicians) were part in the qualitative content analysis to implement investigator triangulation and to ensure the findings’ trustworthiness. Moreover, findings were repeatedly discussed in a multidisciplinary team (comprising pediatricians, infectious disease specialists, and clinical microbiologists).

Although we performed a large number of interviews at two different time periods to collect a wide range of perspectives, some limitations have to be mentioned. First, factors we identified as facilitating or hindering the implementation of the intervention reflect the perspectives of those who were interviewed and may not be generalizable in the sense of quantitative research findings. Second, as data had to be collected in two short time periods, we were not able to perform data analysis while data collection was taking place to consecutively ensure data saturation. We counteracted this by a high number of interviews with purposively selected participants. This allowed us to achieve data saturation, although the great amount of data might have limited the in-depth analysis. In particular, we chose not to focus on changes over time. Rather we adopted a descriptive approach in line with the principles of qualitative content analysis, to highlight practical barriers and facilitators of the implementation of surveillance protocols in pre-school children DCCs. Third, the study was conducted in a low incidence phase, and expected consequences (e.g., quarantine, contact person measures, etc.) occurred only sporadically. Therefore, experiences of these consequences of testing procedures are lacking.

### Meaning and Implication for Practice and Policy

The COVID-19 pandemic impacted heavily on the wellbeing and the daily life of pre-school children and their families. Well-designed surveillance protocols aimed to control the spread of the infection may contribute to reduce this burden and to avoid preventive generalized closures of these facilities. Several recommendations may be drawn from our study to design such interventions in an age-adequate, child-friendly and inclusive manner. First, tests should comprise non-invasive procedures, such as saliva test, and should be performed by trained test teams. Second, children should be approached appreciating their efforts, and accompanying persons should be encouraged in their motivational presence. Third, a transparent, inclusive, and appreciative communication and information strategy addressed to children, their families, and the DCC staff should be adopted to increase acceptance and participation rates. Fourth, motivation to participate in surveillance protocols can be fostered by offering easy access to test procedures and timely results to those directly involved in the surveillance protocols, as well as to their household members or other contact persons. Fifth, concerns about potential negative consequences of the implementation of surveillance protocols, such as quarantines or high workloads, need to be addressed in advance.

### Implication for Future Research

To enhance the credibility and generalizability of our results, the impact of those factors that emerged as potentially influencing the implementation of surveillance protocols in DCCs on participation rates and satisfaction should be studied with quantitative methodologies. Moreover, since we only focused on parents and CCWs, perspectives of children remain unexplored and may be investigated through an ethnographic approach, using direct observations.

## Conclusion

This qualitative study with DCC workers and parents of children who were regularly tested for SARS-CoV-2 infections in nine German DCCs uncovered factors that might facilitate or hinder implementation of surveillance protocols. Comprehensive information, close communication with participants, and attention to children’s wellbeing appear to be critical to implement surveillance protocols effectively. These findings should be taken into account when public health measures are planned to prevent the spread of SARS-CoV-2 or other infectious diseases in facilities where children, their families and workers are affected equally.

## Data Availability Statement

The raw data supporting the conclusions of this article will be made available by the corresponding author, without undue reservation.

## Ethics Statement

The studies involving human participants were reviewed and approved by the Ethics Committee of the University of Würzburg. The patients/participants provided their written informed consent to participate in this study.

## Author Contributions

DG, OK, JL, PH, and IG conceptualized the study. DG, WF, and MK performed interviews. JF, AS, JW, FP, KH, VR, and GE collected data for quantitative analyses. DG, WF, PK, and IG performed data analysis and together with MK, interpreted the results. PK drafted the manuscript. OK and JL acquired funding. All authors revised the manuscript critically and approved the final version.

## Conflict of Interest

The authors declare that the research was conducted in the absence of any commercial or financial relationships that could be construed as a potential conflict of interest.

## Publisher’s Note

All claims expressed in this article are solely those of the authors and do not necessarily represent those of their affiliated organizations, or those of the publisher, the editors and the reviewers. Any product that may be evaluated in this article, or claim that may be made by its manufacturer, is not guaranteed or endorsed by the publisher.
