# Corrigendum: Parents' and childcare workers' perspectives toward SARS-CoV-2 test and surveillance protocols in pre-school children day care centers: A qualitative study within the german Wü-KiTa-CoV project

**DOI:** 10.3389/fmed.2022.973518

**Published:** 2022-07-21

**Authors:** David Gierszewski, Peter Konstantin Kurotschka, Maike Krauthausen, Willi Fröhlich, Johannes Forster, Franziska Pietsch, Andrea Streng, Viktoria Rücker, Julia Wallstabe, Katrin Hartmann, Thomas Jans, Geraldine Engels, Marcel Romanos, Peter Heuschmann, Christoph Härtel, Oliver Kurzai, Johannes Liese, Ildikó Gágyor

**Affiliations:** ^1^Department of General Practice, University Hospital Wuerzburg, Wuerzburg, Germany; ^2^Institute for Hygiene and Microbiology, University of Wuerzburg, Wuerzburg, Germany; ^3^Department of Pediatrics, University Hospital Wuerzburg, Wuerzburg, Germany; ^4^Institute of Clinical Epidemiology and Biometry, University of Wuerzburg, Wuerzburg, Germany; ^5^Clinic and Policlinic for Child and Adolescent Psychiatry, Psychosomatics and Psychotherapy, University Hospital Wuerzburg, Wuerzburg, Germany; ^6^Clinical Trial Center Wuerzburg, University Hospital Wuerzburg, Wuerzburg, Germany; ^7^Leibniz Institute for Natural Product Research and Infection Biology—Hans-Knoell- Institute, Jena, Germany

**Keywords:** parent, childcare worker, child day care centers, child preschool, public health surveillance, COVID-19 testing, qualitative research, interview (MeSH)

In the published article, there was an error in Figure 1 as published. The colors of Figure 1 didn't correspond with the colors in the legend. The corrected Figure 1 and its caption appear below.

**Figure 1:**
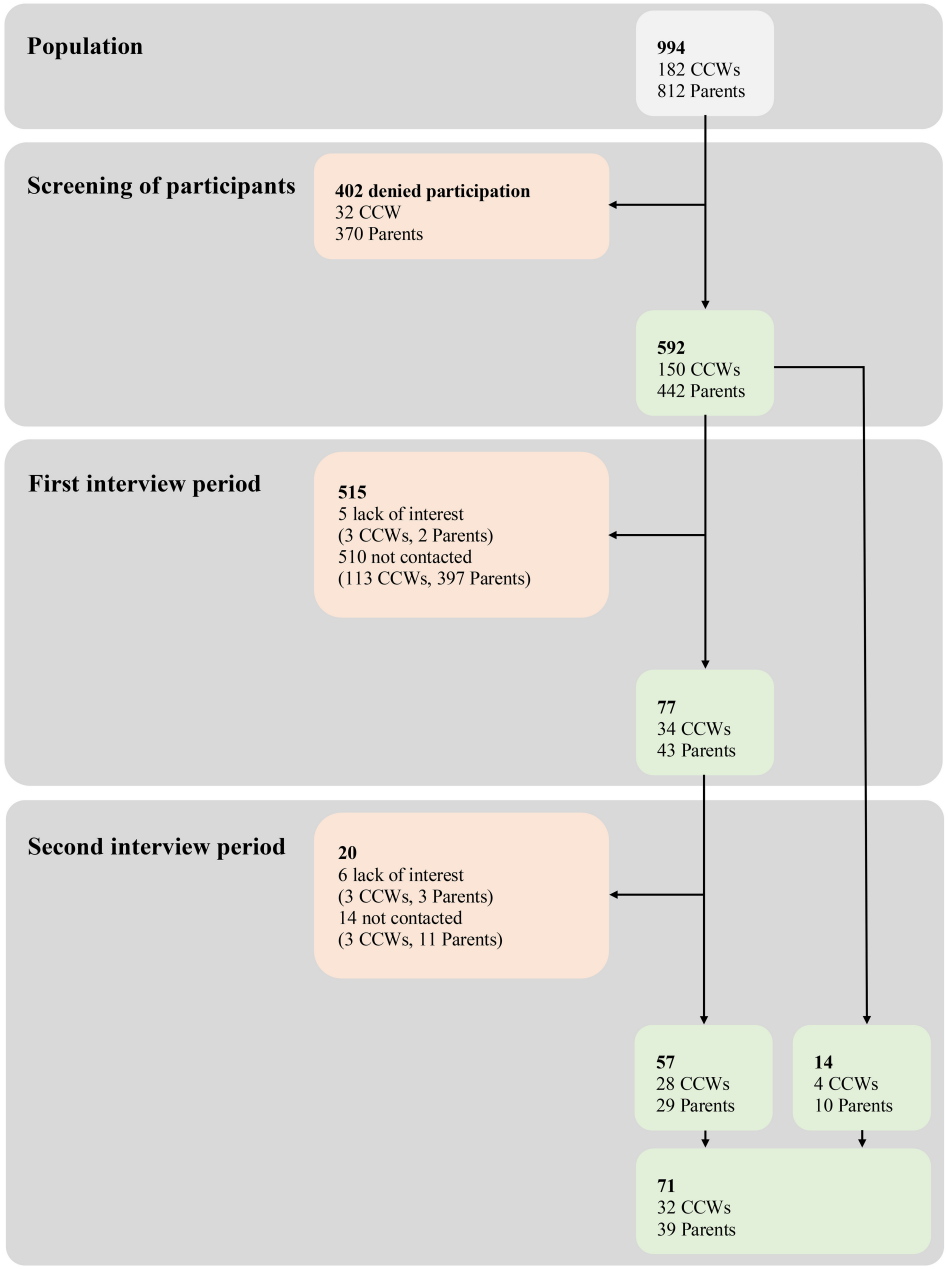
Flow of participants throughout the study with reasons for inclusion/exclusion. Light gray, parents and CCWs belonging to the nine DCCs involved in Wü-KiTa-CoV and screened for interest in being interviewed; light green, parents and CCWs included; light red, parents and CCWs excluded (with reasons).

The authors apologize for this error and state that this does not change the scientific conclusions of the article in any way. The original article has been updated.

## Publisher's note

All claims expressed in this article are solely those of the authors and do not necessarily represent those of their affiliated organizations, or those of the publisher, the editors and the reviewers. Any product that may be evaluated in this article, or claim that may be made by its manufacturer, is not guaranteed or endorsed by the publisher.

