# Supplementary material for: Parents’ and Childcare Workers’ Perspectives Toward SARS-CoV-2 Test and Surveillance Protocols in Pre-school Children Day Care Centers: A Qualitative Study Within the German Wü-KiTa-CoV Project
Source: Front Med (Lausanne). 2022 Apr 29;9:897726. doi: 10.3389/fmed.2022.897726 (PMC9102144; doi:10.3389/fmed.2022.897726)
Supplement: Supplementary file 1 [file Data_Sheet_1.docx]

**Table 1**. Interview guide for the first interview period in the Wü-KiTa-CoV study

**Introduction**

- content and purpose of the study
- procedure of the interview
- the participation in the study is voluntary; non-participation is not linked to any disadvantages and revocation of the consent is possible at any time
- the conversation is audio-recorded on tape and transcribed verbatim under applicable data protection regulations (recording starts after the introduction)

**Opening**

*I will ask you a few questions now. You are welcome to take as much time as you like in answering them and to tell me in detail everything that occurs to you on the subject - even if it seems unimportant to you, just tell me anything that comes to mind.*

**Topic 1: Personal affection**

*How have you experienced the pandemic since the shutdown? Tell us anything that comes to mind regarding this matter.*

- *What impact did the pandemic have on you personally?*
- *on your family life?*
- *on your work?*
- *Have there been any COVID infections in your environment? If yes, how have these infections affected you?*

**Topic 2: Dealing with the pandemic**

*How did you cope with the situation? There are good and bad experiences. Tell us about situations that were not easy or that you found difficult.*

**Topic 3: Socio-political approach**

*What do you think the situation in Germany would look like in a year if the pandemic continues? In your opinion, what would be an optimal situation?*

**Topic 4: Surveillance protocols**

*What are your expectations regarding the surveillance protocols?*

- *What else would you like to see?*
- *Where do you see possible problems?*

**Closing**

*That brings me to the end of my questions for now. However, there still might be something important to you that we have not yet addressed. Is there anything else you would like to add at this point?*

**Participant information**

Gender

□ male □ female □ diverse

Age

□ 18-30 □ 31-40 □ 41-50 □ 51-65 □ >65

Residence (inhabitants)

□ <5k □ 5k - <20k □ 20k - <100k □ >100k

Size of household (people)

□ 1 □ 2 □ 3 □ 4 □ 5 □ 6 □ >6

**Postscript**

**About the interview**

Interview-ID

Interviewer

Date of the interview

Location of the interview

Duration of the interview

**Interview situation (atmosphere)**

**Special occurrences during the interview (course of conversation, disturbances)**

**Striking and emerging topics**

**Table 2**. Interview guide for the second interview period in the Wü-KiTa-CoV study

**Introduction**

- content and purpose of the study
- procedure of the interview
- the participation in the study is voluntary; non-participation is not linked to any disadvantages and revocation of the consent is possible at any time
- the conversation is audio-recorded on tape and transcribed verbatim under applicable data protection regulations (recording starts after the introduction)

**Opening**

*I will ask you a few questions now. You are welcome to take as much time as you like in answering them and to tell me in detail everything that occurs to you on the subject - even if it seems unimportant to you, just tell me anything that comes to mind.*

**Topic 1: Personal affection**

*How have you experienced the pandemic since the first shutdown? Tell us anything that comes to mind regarding this matter.*

- *What impact did the pandemic have on you personally?*
- *on your family life?*
- *on your work?*
- *on your leisure activities?*
- *Have there been any COVID infections in your environment? If yes, how have these infections affected you?*

*You have already been invited for an interview as part of the Wü-KiTa-CoV study once. Today I would like to ask you how you have experienced the further course of the pandemic since the first interview. Tell us anything that comes to mind regarding this matter.*

- *To what extent were there further effects on you personally?*
- *on your family life?*
- *on your work?*
- *on your leisure activities?*
- *Have there been any COVID infections in your environment? If yes, how have these infections affected you?*

**Topic 2: Dealing with the pandemic**

*How did you cope with the situation? There are good and bad experiences. Tell us about situations that were not easy or that you found difficult.*

*How have you coped with the further course of the pandemic since the first interview?*

- *To what extent has your approach to the pandemic changed since the first interview?*
- *Why has it changed? Not changed?*

**Topic 3: Socio-political approach**

*How do you assess the situation in Germany on dealing with the pandemic?*

- *What do you wish for?*

**Topic 4: Situation at daycare centers**

*How have you perceived the situation at the daycare center since the first shutdown?*

- *How do you think the situation at the daycare center has changed over the course of the pandemic?*
- *What impact did the daycare center shutdowns have on you personally?*
- *on your children?*
- *on your family life?*
- *your leisure activities?*
- *How did you perceive the hygiene regulations introduced at the daycare center?*
- *Where have difficulties with the hygiene regulations arisen?*
- *What would you like to see at the daycare center in dealing with the pandemic?*

**Topic 5: Surveillance protocols**

*How did you perceive the surveillance protocols used?*

- *Where have difficulties arisen?*
- *What effects did the surveillance protocols have on the daycare center?*
- *on you personally?*
- *on your children?*
- *How do you estimate an extension of the surveillance protocols used?*

**Closing**

*That brings me to the end of my questions for now. However, there still might be something important to you that we have not yet addressed. Is there anything else you would like to add at this point?*

**Participant information**

Gender

□ male □ female □ diverse

Age

□ 18-30 □ 31-40 □ 41-50 □ 51-65 □ >65

Residence (inhabitants)

□ <5k □ 5k - <20k □ 20k - <100k □ >100k

Size of household (people)

□ 1 □ 2 □ 3 □ 4 □ 5 □ 6 □ >6

**Postscript**

**About the interview**

Interview-ID

Interviewer

Date of the interview

Location of the interview

Duration of the interview

**Interview situation (atmosphere)**

**Special occurrences during the interview (course of conversation, disturbances)**

**Striking and emerging topics**

**Table 3**. **Quotations related to the main topical categories**

| **Main topical category: Generating valuable knowledge** | |
| --- | --- |
| Sub-category | Quotations |
| Generate new data | “I am very open to [the surveillance protocols]. I think it's good that such measures are taken. I'm glad we can help.” (CCW 11, female, interview period 1)  “I think the action is good, because you have to get data somewhere in order to somehow find distributions, patterns, clusters.” (Parent 11, male, interview period 2)  “And yes, people might then more cautious about the pandemic. Aren’t so careless about it. And I think that because of such actions, they might actually think about it.” (CCW 1, female, interview period 1) |
| Avoiding closures of DCCs | “When there will be a pandemic again, another one, that [surveillance protocols] might then be common practice (…) in such large facilities. Because that's the only way to get it under control without having to shut everything down.” (CCW 1, female, interview period 1) |
| Information for policy makers | “That in general from the results of these studies, politics can come up with more specific or more precise restrictions or measures. Which measures are reasonable?” (Parent 12, male, interview period 1) |
| Understand the role of children in the pandemic | “Well, I would say that information is collected on whether the children transmit it or how they transmit it and draw conclusions from that, how the actions in the DCCs and the schools can possibly be adapted to that maybe.” (Parent 13, male, interview period 1) |
| Understand the impact of the pandemic on children | “On the other hand, [by implementing such studies] a little more attention is paid to how this shutdown and this pandemic affect the children.” (Parent 1, female, interview period 2) |
| Skepticism about generating new data | “I don't really expect any scientific findings to come out of [the surveillance protocols], that it will help in any way, I don’t really think so.” (CCW 12, female, interview period 1) |
| Doubts on reliability of tests | “Well, I would just like to find out how good the tests [referring to the surveillance protocols] are in principle.” (Parent 3, female, interview period 1)  “Well, if someone is tested positive, then this person has been among the children for days already. So… is that anyhow useful?” (Parent 14, female, interview period 1)  “I am just not entirely sure whether a COVID-19 disease can actually be diagnosed accurately using this test. I also believe that there is a relatively high number of unreported cases, or false negative results.” (Parent 2, female, interview period 2)  “And I wonder how that [the surveillance protocols] can actually be implemented in the middle of the flu-season.” (CCW 5, male, interview period 1) |
| Low participation rates in the study | “I think that the most important, the most interesting test subjects are not included in the study.” (CCW 2, male, interview period 1)  “(…) because if 50 percent or less [participate], and the others don't, then it’s basically useless.” (Parent 15, female, interview period 2)  “[The reason was] the participation of the people. So people say: ‘Why do I have to test all the time? I don’t accept it.’ So it’s already very difficult to try to decide what kind of vaccination to get. But I just think that many people, I can see it among my own good friends, where I thought: ‘Of course they will have their children participate in that’, […] [but they said:] ‘No, I would never have my child pricked at the DCC.’ For me that goes without saying.” (Parent 3, female, interview period 1)  “I mean, if you could introduce mandatory actions in all facilities in a sensitive way … no idea, excluding non-participants from the DCC or something like that, then this would maybe at least in the context of the DCC increase the awareness of parents and children.” (CCW 2, male, interview period 1) |
| Other testing possibilities | “(…) but I wouldn’t see it as a special benefit anymore (…). Simply because, thank God, (…) the whole test situation has changed. It was a sort of luxury, so to speak, that you could test yourself so quickly, but that's no longer the case.” (CCW 3, female, interview period 2) |
| **Main topical category: Impact on daily life** | |
| Sub-category | Quotations |
| Fear of increased workload | “(…) it is of course a huge logistical effort, which of course does not leave the everyday work unaffected. When you have four teams in the facility that have to do the nasal swab early in the mornings [before they can start their work].” (CCW 10, male, interview period 1) |
| Smooth integration in daily routines at the DCCs and at home | “(…) [It] had little impact on our everyday pedagogical work. It was just a common ritual, once per week they were here, the team was here.“ (CCW 4, female, interview period 2)  “It was completely integrated in the daily routine. Coming into the facility, taking off jacket and shoes, washing hands, putting down the backpack in the office, going to the gym, doing the nasal swab, checking e-mails.” (CCW 10, male, interview period 2)  “Of course I was a bit skeptical at the beginning, I thought, oh (…) that will be additional work (…). But in the end it really wasn’t, because you really had very little work with it. So I can only say that in the end it was actually a positive experience for us as a team (…).“ (CCW 3, female, interview period 2) |
| Need to travel to test centers for PCR testing | “Or I didn't want any appointments. Additional appointments where you have to go somewhere.“ (Parent 16, female, interview period 1)  “Of course, it would be good if there was another option [to get tested] for people who just have a long way to go. (...) So that when there are several cases or a suspected case, the test can basically take place right there in the DCC.” (CCW 13, male, interview period 1) |
| Quickly delivered test results | “I expect that there may be difficulties in actually implementing this: when there are three colleagues in one group [of children] and two of them have cold symptoms [and] one gets tested, that the second one maybe doesn’t want to leave the third alone, feeling like ‘who should be responsible for the group’.” (CCW 5, male, interview period 1)  “I only heard about it from colleagues who had been tested, which was quite ok, because you knew within 24 hours, how you could plan, how you should work. So it was a relief for us in a way. And also the possibility, to keep it in the back of your mind, that if something happens, then I will get the test result relatively fast or get the test result fast and don’t have to, don’t have a long downtime.“ (CCW 20, male, interview period 2) |
| Fear of quarantines in case of positive test results | “I really worried that one day the saliva sample might lead to a positive result and then the DCC would be closed for two weeks, which would have been relatively difficult to manage for both of us working parents.” (Parent 2, female, interview period 2)  “So we would no longer get us tested voluntarily (…) because we do NOT want to be in quarantine a third time and, and, and…” (CCW 14, female, interview period 2) |
| Carelessness of the testing | “So it would be a problem if the parents (…) don't take the matter seriously (…) because then the result might simply be wrong or there might be no result at all.” (Parent 17, female, interview period 1)  “I noticed that some colleagues gargled with children because their parents either didn't manage to do it at home or forgot it.“ (CCW 6, female, interview period 2) |
| **Main topical category: Communication and information** | |
| Sub-category | Quotations |
| Transparent communication | “The phone numbers were in the leaflet if you had any questions, and a team came by regularly. (...) I think this actually shows that this way parents could build trust.” (CCW 6, female, interview period 1)  “That was only at the beginning. At the beginning we had this discussion, where of course some of them, pandemic deniers, were still there. I have told you about this mother. That was sometimes a big brouhaha. And then, that some people didn’t understand that was voluntary. They immediately said ‘You cannot force us’. That was something that was really difficult. But that settled after a while anyway. Apart from that everything went well.” (CCW 1, female, interview period 2) |
| Engagement with critical participants | “The only problem is, that some parents don’t want to support it. But that’s also a bit, a bit about rooting for it. But there is, I think you see that everywhere right now, that there are these critical voices and even denying voices and that you have to somehow try to persuade them. (…) Even people who you wouldn’t have, wouldn’t have expected it before, to think that way. So there are often these surprising comments by parents.“ (CCW 13, male, interview period 1) |
| Cooperation on equal terms | “Yes, it went really well in the cooperation with the team, I have to say, the director said so as well, that there was basically this cooperation and that was just positive. (…) Also I have to say, it was always interesting to participate in this thing. I have to say, I was skeptical at the beginning, I didn’t really want to participate, you know. And then I was happy I had participated.” (CCW 12, female, interview period 2) |
| No possibility of consent withdrawal of sample utilization | “(…) When you signed up for it, it said somewhere in the documents that the samples provided would also be used for other purposes. And honestly I didn't think that was so good, that first of all you didn't have the opportunity to contradict that, maybe not everyone would like that, and that you didn't even know about what would happen with these samples later on. So that I found something like ‘I see, okay.’ (laughs) A bit non-transparent maybe.” (Parent 4, female, interview period 2) |
| Prompt, brief and thorough information about the surveillance protocols | “Well, you were always kept up to date through the e-mails. I thought that was very good. Whenever something has changed.” (Parent 18, female, interview period 2) |
| Too much information about the surveillance protocols | “(...) so that it is more condensed, on single sheet in A4 format, summarized very briefly, in two sentences, what do you want with this study? What happens when?” (CCW 7, female, interview period 1) |
| Not being informed about the overall results of the surveillance protocols | “And I just don't know, I mean, I don't know how effective that is either. I also don't know if we receive an overview of how things went in the other facilities or with the other tests.” (Parent 5, female, interview period 2) |
| **Main topical category: Children's wellbeing** | |
| Sub-category | Quotations |
| Fear of development of an aversion to the DDC and medical procedures | “(…) because I’m worried that the children are no longer happy to come to the DCC or that they would be afraid of the swabbing, because I think they [referring to the tests] are quite uncomfortable.” (CCW 15, female, interview period 2)  “(…) That because of this [referring to swabs], I could imagine, an antipathy or anti-attitude towards all doctors could develop.” (Parent 16, female, interview period 1) |
| Young age of the child | “I am now in the nursery for children aged 1 to 3 (…) most kids just can't spit it out or are not able to keep it for those 10 seconds in their mouth. So, whether the study is feasible then... But I think older children can do it.” (CCW 8, female, interview period 1)  “I would have done the nasal swab if he was older.” (Parent 19, female, interview period 2) |
| Fear of unpleasant feelings | “But when the child says: ‘I no longer go to the DCC, because I get a nasal swab and it hurts’.“ (CCW 4, female, interview period 1) |
| Invasive procedures (i. e. midturbinate nasal swab) | “We consciously, consciously, decided against having this nose swab done weekly (...). But afterwards (...) we would have done it differently. Now that I know it wasn't that harmful.” (Parent 6, female, interview period 2) |
| Testing in the home environment | “(…) that I can just do it myself at home [referring to salivary tests]. (…) Because my children are very sensitive and fearful. So, they might even do it voluntarily, but I think it's easier in the home environment.“ (Parent 2, female, interview period 1) |
| Tests performed next to a parent | “Of course that depends very much on the age. With the older ones you can talk about it. They understand it. With the one- or two-years old ones that is a bit tricky, I think. But well. No. I think that children are also resilient. I don’t really expect bad things to happen. Especially as the parents are there too.” (CCW 7, female, interview period 1) |
| Motivation through parents | “Sometimes he didn't feel like it, but then let himself be persuaded.“ (Parent 7, female, interview period 2) |
| Age adequate/Child-friendly explanations | “I just explained to him that it was like taking a booger out of his nose.“ (Parent 7, female, interview period 1) |
| Changing test teams | “It was a pity that the testing staff changed almost every time. I think it would have been easier for the children if there would have been less changes.” (CCW 9, female, interview period 2) |
| Child-friendly testing environment | “[It is important] that they tell to the children how great they are (…). And that they are actually helping everyone with it. That it is courageous and important and yes, like that.“ (CCW 9, female, interview period 1)  “Well, the children were always happy to be able to hand in their samples and to get a stamp and a sticker here and were actually more interested in what was actually going on.“ (CCW 16, male, interview period 2)  “I think that the children experience themselves as doing something for the community. And that they are proud.” (CCW 9, female, interview period 2) |
| **Main topical category: Sense of security** | |
| Sub-category | Quotations |
| Protocols as sensitive indicators of epidemic outbreaks | “Because I’m simply hoping for some kind of security (…) for the staff, children and parents. That’s to say, I can control the local outbreak situation, I can be responsive to it and can send them, when they are not even sick yet, in quarantine.” (CCW 10, male, interview period 1)  “I basically found it good, the testing (…), because we had (…) at least once per week the verification, regarding (…) one family member, if there was an infection. And this is how I always saw it in the end, that if one of us somehow was infected with COVID, then it would most probably relatively fast spread within the whole family, because we are all pretty close here, like in a house or a household, so to say.” (Parent 8, male, interview period 2) |
| Sense of protection at work | “I see it primarily as a protection for us as CCWs, but also to some degree for my family (…).“ (CCW 12, female, interview period 1) |
| Less fear to transmit the infection to other people | “So you basically felt more safe, not with regard to yourself, but when we had contact with family, friends, when you met someone after all.” (Parent 9, male, interview period 2) |
| Having someone to discuss concerns in case of symptoms | “That was just a relief for me. To know that there is a contact person.” (CCW 17, female, interview period 2) |
| Opportunity to get tested timely without complications | “(…) That I can simply take a test quickly and don't have to call somewhere and then wait and wait four days for the result.” (CCW, female, interview period 2)  “I mean, right now, during winter time, you often have a dry throat or easily catch a cold and so on and during the situation as it is right now you will immediately worry about it.” (CCW 19, male, interview period 2) |
| Opportunity to get tests for household members | “We were then even able to have our [older] children who were at school tested at the DCC. That was great.” (Parent 10, female, interview period 2) |
| Fear of increased risk of infections due to alternating testing staff | “(…) That the doctors and the teams that come in infect the children sick. Because you don't know where they were before.” (CCW 10, male, interview period 1) |
